# WNT Signaling in Hematological Malignancies

**DOI:** 10.3389/fonc.2020.615190

**Published:** 2020-12-21

**Authors:** Michela Frenquelli, Giovanni Tonon

**Affiliations:** ^1^ B-cell Neoplasia Unit, Division of Experimental Oncology, IRCCS San Raffaele Scientific Institute, Milan, Italy; ^2^ Functional Genomics of Cancer Unit, Division of Experimental Oncology, IRCCS San Raffaele Scientific Institute, Milan, Italy; ^3^ Center for Omics Sciences (COSR), IRCCS San Raffaele Scientific Institute, Milan, Italy

**Keywords:** multiple myeloma, WNT, ROR2, Wnt/b-catenin, microenvironment

## Abstract

The role of the WNT signaling pathway in key cellular processes, such as cell proliferation, differentiation and migration is well documented. WNT signaling cascade is initiated by the interaction of WNT ligands with receptors belonging to the Frizzled family, and/or the ROR1/ROR2 and RYK families. The downstream signaling cascade results in the activation of the canonical β-catenin dependent pathway, ultimately leading to transcriptional control of cell proliferation, or the non-canonical pathway, mainly acting on cell migration and cell polarity. The high level of expression of both WNT ligands and WNT receptors in cancer cells and in the surrounding microenvironment suggests that WNT may represent a central conduit of interactions between tumor cells and microenviroment. In this review we will focus on WNT pathways deregulation in hematological cancers, both at the ligand and receptor levels. We will review available literature regarding both the classical β-catenin dependent pathway as well as the non-canonical pathway, with particular emphasis on the possible exploitation of WNT aberrant activation as a therapeutic target, a notion supported by preclinical data.

## Introduction

The WNT signaling pathway is central for development and homeostasis within tissues. The WNT signaling cascade is initiated by the interaction of WNT proteins (lipid-modified secreted glycoproteins) with various receptors and co-receptors whose activation elicit several processes, such as cell proliferation, differentiation, apoptosis, polarity, migration and invasion (1, 2).

WNT proteins are evolutionary conserved and in mammals WNT family genes comprise 19 members. These secreted, cystein-rich proteins exert their effects through the interaction with the Frizzled family of proteins (consisting of 10 members). Frizzled (FZD) are seven-pass transmembrane, and each FZD protein contains a cysteine-rich domain (CRD) with approximately 10 cysteine residues, essential for the binding to WNT proteins (3, 4).

Apart from FZD, other proteins are involved in WNT recognition: the co-receptor low-density lipoprotein receptor-related proteins (LRP 5 and 6), the receptor Tyr kinase-like orphan receptor family (ROR1 and ROR2), the Receptor Tyrosine Kinase (RYK) and the protein Tyr kinase 7 (PTK7).

The WNT pathway is broadly divided into canonical (β-catenin-dependent) and non-canonical (β-catenin-independent), depending on the agonists, the receptors and the intracellular players involved. Given the high number of both WNT ligands and receptors, the possible combinations are numerous, making its regulation exceedingly complex. The activation of either signaling pathway is receptor and ligand dependent and yet is also influenced by the cellular context. As an approximation, certain WNTs (WNT1, WNT3A and WNT8) activate the canonical β-catenin-dependent WNT signaling pathway by preferentially binding to FZD receptor coupled to LRP5/6 co-receptors. Conversely, WNT5A and WNT11 preferentially bind to ROR1, ROR2 and RYK receptors and are predominantly involved in the β-catenin-independent, non-canonical pathway.

Irrespectively from the signaling branch activated upon binding of WNT ligands to FZD or other receptors, the signal is commonly transduced to a protein called Disheveled (DVL) that represents the molecular hub executing both the canonical and non-canonical pathways (5). How can the same DVL protein be involved in different signaling pathways? The molecular wiring underlying this behaviour remains unknown, but various mechanisms have been proposed to confer specificity to DVL activity, including different phosphorylation events on DVL or the establishment of alternative interactions between DVL and its protein partners. This latter hypothesis is supported by the presence of three conserved domains in DVL protein (namely DIX, DEP and PDZ) which mediate its interaction with effector proteins involved in different signaling branches. Indeed, it has been suggested that DIX and PDZ domains are implicated in the activation of β-catenin signaling, whereas DEP and PDZ are preferentially engaged in the interactions with proteins (e.g. Daam1, Prickle) that trigger the non-canonical signaling pathway (6).

## WNT/β-Catenin Signaling

As mentioned earlier, FZD proteins act as main receptors for WNT ligands, together with the co-receptor LRP5/6.

**Figure 1:**
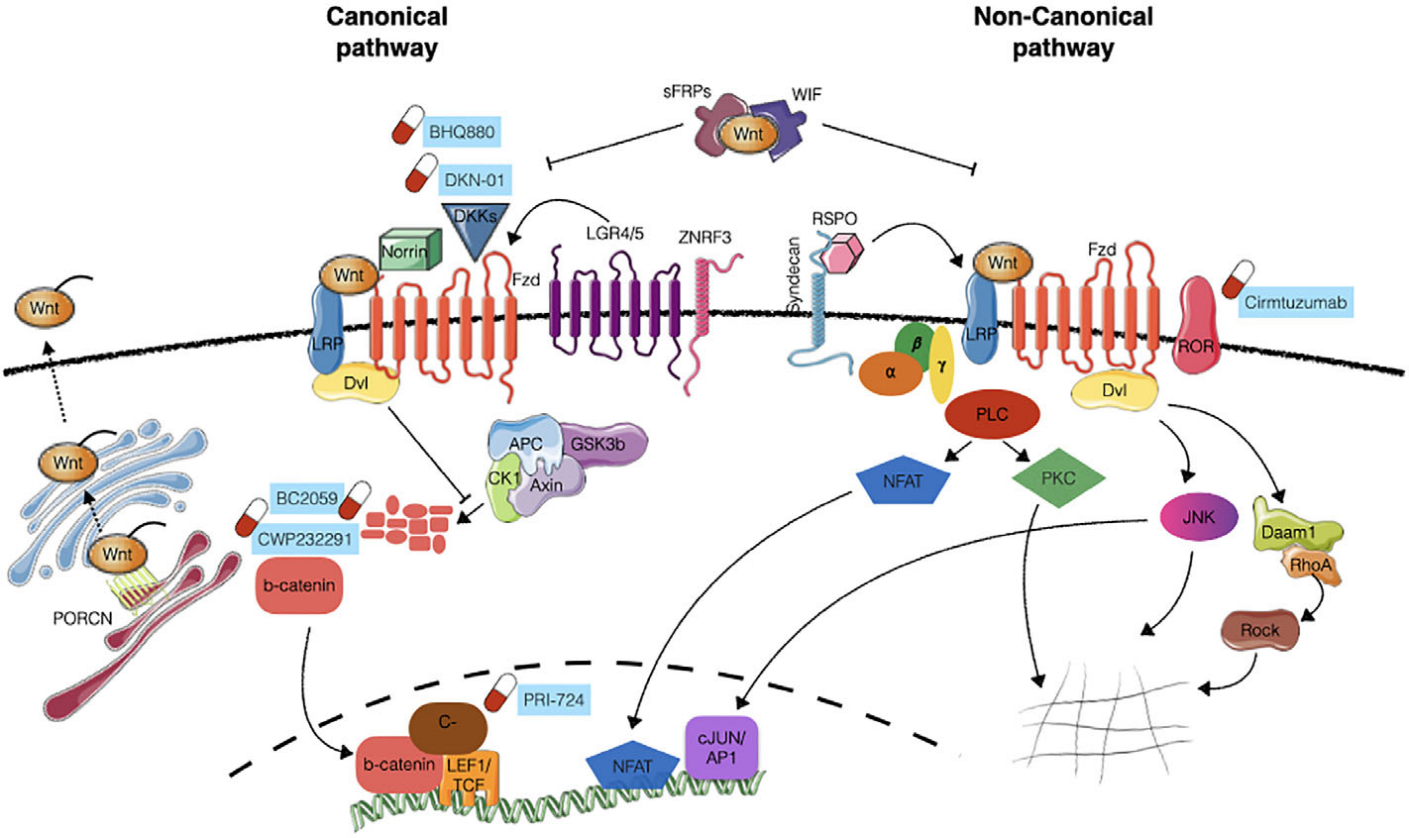
WNT pathaway signaling. Schematic representation of canonical (β-catenin dependent) and non-canonical (β-catenin independent) WNT signaling. WNT ligand interaction with FZD receptors together with LRP5/6 co-receptors inhibits the activity of the “destruction complex” (composed by Axin, APC, CK1 and GSK3β), allowing the stabilization of β-catenin, its translocation into the nucleus and the transcriptional activation of target genes. The activation of the non-canonical pathway involves additional co-receptors (e.g. ROR1 and ROR2) and is mediated by small GTPases RhoA, Daam1 and ROCK, acting on cytoskeleton remodelling, or PLC, JNK and NFAT, acting on transcriptional activity. Further layers of regulation of the pathway are exerted at the level of WNT ligands modification/secretion (mainly through PRCN), engagement of additional activator proteins (Norrin, Syndecans, RSPO, LGR4/5, ZNRF3) or the presence of antagonists (DKK, WIF, sFRPs). Compounds under clinical evaluation are indicated next to the appropriate target. Images have been created with Servier Medical Art (https://smart.servier.com/).

In the absence of exogenous WNT, the pathway is maintained in the “off” state by the so-called “β-catenin destruction complex”. This multiprotein complex, composed by Axin, β-catenin, APC, GSK3 and CK1, keeps β-catenin at low levels by phosphorylating it, and thus marking it for recognition by β-TRCP ubiquitin ligase, leading to its subsequent ubitiquination and degradation (7, 8). When a WNT molecule binds to FZD, it induces the phosphorylation of LRP6 (9), and the association of DVL proteins to the receptor. Both these events that are required for initiation of the signaling cascade resulting in the exposure of DVL’s DIX domain that works as a docking site for Axin pulling it away from the destruction complex. Moreover, the cytoplasmic tail of LRP5/6 contains several PPPSPxS domains that can bind Axin and become a competing substrate for GSK3. As a result, the destruction complex is disassembled and β-catenin is no longer marked for destruction. Therefore, β-catenin accumulates in the cytoplasm and then translocates in the nucleus (10), where it associates with the TCF/LEF transcription factors and activates the transcription of target genes such as Myc, Cyclin D1, Survivin and MMP (11) mainly implicated in the regulation of cell proliferation (**Figure 1**).

## Non-Canonical WNT Signaling

The interactions of other WNT ligands (such as WNT5A and WNT11) and receptors (including ROR1 and ROR2) results in the activation of alternative signaling routes different from the canonical β-catenin dependent pathway, known as WNT non-canonical pathways. The first example of a non-canonical pathway is the Planar Cell Polarity (PCP)/Jun N-terminal kinase (JNK) signaling cascade that involves the activation of JNK, Rho, Rac and Rho kinase (ROCK) proteins (12). In this context, WNT ligand binding to FZD receptor stimulates the recruitment of DVL, which contrarily to what happens in the canonical-pathway, interacts with DAAM1 (Disheveled associated activator of morphogenesis 1) thereby stimulating Rac and Rho small GTPases and JNK, whose activation results in actin polymerization and cytoskeletal modifications (13) (**Figure 1**). In addition to the PCP pathway, another significant noncanonical pathway is the WNT/Ca^2+^ pathway, characterized by an increase in the intracellular calcium level as a result of WNT binding. This pathway subsequently activates heterotrimeric G proteins and Phospholipase-C (PLC) which hydrolyses membrane phospholipids to di-acyl glycerol and inositol 1,4,5-triphosphate (IP3). This neo-generated IP3 causes the release of calcium from the endoplasmic reticulum which activates Protein kinase C (PKC) and ultimately activating calcineurin and NFAT transcription factor, thus regulating transcriptional programs involved in cell fate and cell migration (14) (**Figure 1**).

## Regulation of WNT Signaling by Additional Agonists/Antagonists

The fine-tuning of WNT signaling activity is also determined by additional proteins that can either stimulate or inhibit WNT pathway. Two major families of growth factors are known to act as WNT agonists: Norrin, and R-spondins (RSPOs). As for the antagonists they can be broadly divided in two categories: the one interfering with WNT ligand (Secreted frizzled-related proteins- sFPRs, Wnt inhibitory factor- WIF) or other binding to the receptor complex (Dickkopf-related proteins- DKKs) (15).

*WNT agonists*: R-Spondin family of growth factors consists of 4 members (RSPO1 to 4), acting on both canonical (β-catenin dependent) and non-canonical signaling. Their mechanism of action is still controversial but it is suggested that RSPO proteins bind to leuchin-rich G protein 4 and 5 (LRG-4 and 5) to sequester ZNRF3, an E3 ubiquitin ligase involved in the turn-over of Fz-LRP6 receptor complex. By sequestering ZNRF3, RSPO-LRG interaction induces the accumulation of the receptor complex on the cell surface, thus enhancing WNT signaling (16). RSPOs can also amplify non-canonical PCP signaling, likely through the interaction of RSPO/Syndecans with WNT/Fz (17).

Another secreted factor capable of enhancing WNT activation is Norrin which was described to interact with Fz4 (18, 19) and to specifically activate canonical signaling.

*WNT antagonists:* The largest family of secreted WNT inhibitors is represented by sFRPs. In humans, it comprises five members (sFRP1-5), all sharing a conserved CRD at the N-terminus. They act binding WNT ligands and preventing their interaction with the receptor, although mouse sFRP3 has been shown to interact with FZD8 (20), suggesting an additional mode of inhibition (21). They potentially bind to different WNT proteins, so they can inhibit both canonical and non-canonical signaling.

WNT Inhibitor factor 1 (WIF-1) is another secreted factor that acts binding to WNT ligands and preventing their interaction with the receptors. In this way, WIF-1 can inhibit both canonical and non-canonical signaling.

Lastly, the DKK family includes four members (DKK1 to 4), with DDK1, 2 and 4 binding to LRP5/6 and inhibiting the canonical β-catenin pathway. DDK3 does not seem to bind to LRP5/6 and is likely acting on a different pathway such as the TGF-β one (22).

## An Additional Layer of Regulation: Secretion of WNT Ligands

WNTS are secreted proteins exerting their function on a short- or long-range gradient in the neighboring cells. Their proper secretion and release in the extracellular space represents an additional level of signaling modulation. Indeed, WNT proteins need indeed to be posttranslational modified in order to be exported and to be biologically active. One of the most crucial modification is acylation, critical for the transportation from the Golgi to the cell surface (23) and for the correct and efficient binding to FZD receptors (24). WNT acylation occurs through the concerted action of three enzymes: stearoyl CoA desaturase (SCD), porcupine (PORCN) and Notum. Of these enzymes, the most studied is PORCN, a membrane-bound O-acyltransferase that acylates WNT molecules at specific sites. Given the prevalence of WNT signaling dysregulation in cancer, the search for drugs that specifically inhibit PORCN represents a possible treatment in those malignancies where WNT signaling is altered (25).

## WNT Signaling in Hematological Cancer

The high level of expression of both WNT ligands and WNT receptors in cancer suggests that WNT may represent a central conduit of interactions between tumor cells and the microenvironment. Although most of the studies of WNT involvement in cancer come from solid tumors, WNT signaling deregulation has also been observed in hematological cancers.

WNT ligands are normally expressed in the hematopoietic stem cells (HSC) compartment and the surrounding microenvironment, and WNT signaling is crucial for HSC self-renewal and homeostasis (26), as well as for the maturation of hematopoietic progenitors (27). The normal hemopoiesis is maintained through the balance between extracellular factors (WNT ligands, agonists and antagonists), cell surface receptors, cytoplasmic components (adapters, destruction complex components etc.) and nuclear factors. Deregulation in any of the involved partners involved could cause hematopoietic disorders.

Aberrant WNT signaling has been reported for example in acute myeloid leukemia (AML). Common chromosomal translocations found in AML (AML1–ETO, PML–RARα and MLL–AF9) result in the stimulation of WNT signaling in AML cells (28, 29), inducing expression of WNT target genes and increasing proliferation. Moreover hyper-activation of the WNT pathway has also been associated to the overexpression of FZD4 (30) and pakoglobin (31). Other evidences suggest that modulation of endogenous WNT antagonists, such as DKK1 and DKK2 (32, 33) or SFRP (34) in AML patients increase the activity of WNT pathway and correlates with adverse clinical outcome.

In chronic myelogenous leukaemia (CML), increased levels of nuclear β- catenin were detected, thus enhancing the self-renewal capacity of CML cells both *in vitro* (35) and in animal models (36). Both canonical and non-canonical WNT signaling seem to be involved in the resistance to the tyrosine-kinase inhibitor Imatinib and in CML relapse as suggested by preclinical data on CML mouse models (37, 38).

WNT pathway deregulation is suggested to exert a crucial role in acute lymphoblastic leukemias (ALL) as well. In T-ALL, that is strictly dependent on Notch pathway alterations, a pivotal role for canonical WNT signaling has been postulated, based on the high expression of β-catenin, its cofactor LEF1 (39) and on the increased proliferation observed in cells expressing high levels of LEF1 (40). In B-ALL, elevated levels of WNT16 have been found in E2A-PBX1 translocated leukemias (41). Moreover, WNT2B, WNT5A, WNT10B and WNT16B ligands, FZD7 and FZD8 receptors and LRP5/6 co-receptors are overexpressed at different levels in B-ALL (42).

Aberrant WNT signaling is not only involved in the development of leukemias originating from the stem cell compartments, but it is also observed in disorders originating from mature cells.

Dysregulated WNT signaling has also been implicated in the development of Multiple Myeloma (MM). Indeed, β- catenin is often overexpressed and constitutively activated in MM cells, impacting on cell proliferation (43). Moreover, different WNT ligands are expressed in the bone-marrow microenvironment of MM patients, acting both in an autocrine and paracrine manner (44). As a result of these alterations, activation of both the canonical and non-canonical pathways has been observed, hence modulating proliferation capacity (45), migration/invasion (46, 47) and resistance to therapy (48). In this frame, we have recently shown that overexpression of the ROR2 receptor mediates myeloma cells interactions with the bone marrow and its depletion *in vivo* results in detachment of myeloma cells from their niche and delays disease progression. We also demonstrated, using *in vitro* and *ex vivo* 3D-culture systems, that ROR2 exerts a pivotal role in the adhesion of cancer cells to the microenvironment, mainly through the PI3K-AKT pathway, and that genetic and pharmacological inhibition of the AKT pathway is able to reduce ROR2-induced adhesion of malignant cells to bone marrow components (46). Deregulation of WNT pathway, also in terms of WNT antagonist expression, such SFRP2 and DKK1, also has relevant effects on the shaping of bone-marrow niche microenvironment, contributing to alter bone homeostasis and resulting in osteolytic bone disease (49, 50).

In chronic lymphocytic leukemia (CLL) several components of the WNT pathway have been found deregulated: WNT3, WNT5B, WNT6, WNT10A, WNT14, and WNT16, as well as the WNT receptor FZD3, are highly expressed in CLL when compared with normal B cells (51–53). Moreover, soluble inhibitors of the WNT pathway (DKKs and SFRPs) are downregulated (54, 55), further potentiating WNT signaling. More importantly the ROR1 receptor is specifically expressed in malignant CLL cells and not in normal B cells and has been implicated in both proliferation and migration of CLL cells (56, 57).

Aberrant WNT signaling can also be found also in lymphomas. Indeed, elevated levels of nuclear β-catenin have been detected in primary samples of different lymphoma sub-types, even in the absence of mutations in APC or β-catenin (58–60), suggesting that the pathway is activated by autocrine or paracrine mechanisms. Indeed, many WNT components are dysregulated in different type of lymphoma. For example, in Mantle Cell lymphoma (MCL) gene expression profiling studies revealed increased expression of FZD7, APC, LRP5, AXIN1and DVL3 (61).

## Discussion

### Targeting WNT Pathway: Is This the Way?

Many players of both the canonical and non-canonical WNT signaling are deregulated in hematological cancers, making the WNT pathway an interesting therapeutic opportunity for blood cancers. In this context, candidate drugs can act on WNT signaling pathway at different levels. The ideal druggable targets are both WNT ligands and the receptors or co-receptors, as they are easily accessible for a small compound. However, also intracellular mediators at different signaling levels or additional regulators of WNT pathways (extracellular regulators like DKKs or sFRPs or regulators of WNT secretion like PORCN) have been proposed as possible targets. In this section we report the main therapeutic agents targeting the WNT pathway and currently under clinical trials for the treatment of hematological malignancies (https://clinicaltrials.gov/, summarized in **Table 1**).

**Table 1:** WNT pathway targeting under clinical investigation.

Drug	Target- mechanism of action	Stage of drug development	Clinical trial identifier	Disease
**Cirmtuzumab**	Anti -ROR1 monoclonal antibody	Phase 1	NCT02222688 NCT02860676	CLL
**Cirmtuzumab/Cirmtuzumab+Ibrutinib**	Anti -ROR1 monoclonal antibody+ BTK inhibitor	Phase 1b/2	NCT03088878	CLL, SLL, MCL
**Cirmtuzumab/Cirmtuzumab+Venetoclax**	Anti -ROR1 monoclonal antibody+ Bcl2 inhibitor	Phase 2	NCT04501939	CLL
**ROR1R-CAR-T Cell Infusion**	Killing of ROR1+ cells	Phase 1	NCT02194374	CLL, SLL
**VLS-101**	Use ROR1 antibody to recognize malignant cells, then killed by the drug MMAE	Phase 1	NCT03833180	CLL, MCL, FL, MZL, DLBCL, RS, BL, LL, T-NHL, ALL, AML, WM
**PRI-724**	Disrupt the interaction of β-catenin and CBP	Phase 1/2	NCT01606579	AML, CML
**CWP232291**	Inhibits β-catenin transcriptional activity	Phase 1	NCT01398462	AML, CML, Myelodiplastic syndrome, Myelofibrosis
**CWP232291/CWP232291+Lenalidomide and Dexamethasone**	Inhibits β-catenin transcriptional activity+ Immunomodulators	Phase 1a/1b	NCT02426723	MM
**CWP232291/CWP232291+ ara-C/cytarabine**	Inhibits β-catenin transcriptional activity	Phase 1/2	NCT03055286	AML
**AEB071**	PKC inhibitor	Phase 1	NCT01402440	DLBCL
**AEB071/AEB071+** **Everolimus**	PKC inhibitor+ immunosuppressor	Phase 1	NCT01854606	DLBCL
**AEB071**	PKC inhibitor	Phase 2	NCT02285244	PML, MCL, SLL, CLL, RS
**DKN-01**	Inhibits DKK1	Phase 1	NCT01457417	MM
**DKN-01/DKN-01+ Lenalidomide/Dexamethasone**	Inhibits DKK1	Phase 1	NCT01711671	MM
**BHQ880+ Bortezomib and dexamethasone**	Monoclonal antibody to DKK1	Phase 2	NCT01337752	MM
**BHQ880/BHQ880+ zoledronic acid**	Monoclonal antibody to DKK1	Phase 1	NCT00741377	MM
**BHQ880**	Monoclonal antibody to DKK1	Phase 2	NCT01302886	Smoldering MM
**Dendritic cell DKK1 vaccine**	DKK1	Early Phase 1	NCT03591614	MGUS, Smoldering MM, MM

CLL, Chronic Lymphocytic Leukemia; MCL, Mantle Cell Lymphoma; FL, Follicular Lymphoma; MZL, Marginal Zone Lymphoma; DLBCL, Diffuse Large B-cell Lymphoma; RS, Richter Syndrome; BL, Burkitt Lymphoma; LL, Lymphoplasmacytoid Lymphoma; T-NHL, T-cell Non-Hodgkin Lymphoma; ALL, Acute Lymphoid Leukemia; AML, Acute Myeloid Leukemia; WM, Waldenstrom Macroglobulinemia; SLL, Small Lymphocytic Leukemia; PML, Pro-myelocytic Leukemia; MM, Multiple Myeloma; MGUS, Monoclonal Gammopathy on unknown significance.

The most advanced clinical application of inhibitors of the WNT family is represented by the monoclonal antibody Cirmtuzumab, targeting the WNT receptor ROR1, that is overexpressed in CLL. Preclinical studies performed with Cirmtuzumab have shown excellent results in terms of induction of apoptosis and inhibition of cell migration both in *in vitro* and *in vivo* models of CLL (62, 63) and phase I clinical trial (NCT02860676) with this monoclonal antibody confirmed its safety and biological activity of Cirmtuzumab, even if a clear clinical benefit was not evident (64). Other clinical trials aimed to test the efficacy of this promising compound in different combination settings are currently ongoing (NCT03088878 Phase 1b/II clinical trial in combination with Ibrutinib) or planned (NCT04501939 phase II trial in combination with Venetoclax).

Promising results were also obtained with PRI-724/C-82 compound, a C-EBP/β-catenin inhibitor, on different models of leukemia (including T-ALL, AML and CML) also in the context of drug resistant cells (37, 65). A phase 1/2 clinical study with this candidate drug (NCT01606579) has been performed on AML and CML patients has been conducted, although the results are not available yet.

Another interesting molecule that impacts on WNT-β-catenin signaling is the CWP232291 inhibitor. This molecule targets β -catenin for degradation, thus inhibiting its signaling and exerting anti-apoptotic and anti-proliferative activities. CWP232291 is currently in use in different clinical trials in AMLs and MM, and it was recently reported that this compound is well tolerated and shows encouraging clinical activity (66).

Moreover, another β-catenin inhibitor, BC2059, was shown to reduce proliferation and induce apoptosis on both MM primary samples and cell lines, alone or in combination with proteasome inhibitors, and to delay tumor growth *in vivo* (67).

As previously mentioned, it is possible to interfere with WNT signaling activation also by targeting extracellular regulators of the pathway. The example of DKK1, that has been exploited as therapeutic target especially in MM, is paradigmatic of how the WNT pathway may represent a real communication route between cells of the microenvironment and tumor cells. Indeed, MM-secreted DKK1 is indeed mainly active on osteoblasts, preventing their differentiation and contributing to the development of osteolytic lesions. The targeting of DKK1 could be achieved through the treatment with the DKN-01 inhibitor, in a phase I trial (NCT01711671 and NCT01457417) or with the anti-DKK1 monoclonal antibody BHQ880, which has been included in an already completed phase 1 (NCT01302886) and phase 2 trials (NCT01337752 and NCT00741377) showing potential clinical activity in myeloma patients (68). DKK1 has also been exploited in an immunotherapy setting in a myeloma mouse model (69) and also with primary myeloma samples (70), and an early phase 1 study (NCT03591614) is going to start soon.

## Conclusions

In this review we tried to focus on the specific aberrations affecting the WNT pathway in different hematological cancer and how these aberrations could be exploited as therapeutic targets, developing drugs and compounds with specific effects on that particular biological process. If on the one hand the presence of many WNT components (19 WNT ligands and more than 15 receptors) offers several possibilities of tackling the pathway (though the problem of redundancy still remains), on the other hand the fact that WNT signaling is essential for many processes in development and homeostasis poses several challenges on the side of drug specificity and safety. The considerable bulk of knowledge accrued so far and the initial testing of several compounds impinging on this pathway suggest that the WNT network is both a crucial mediator of cancer cell survival and proliferation as well as an enticing target for new drugs. Nevertheless, much effort is still active on the identification of peculiar abnormalities and on the way to specifically target them, with the final aim of exploiting the WNT pathway alterations to stratify patients and provide novel tools to be exploited in a personalized medicine setting.

## AUTHOR CONTRIBUTIONS

MF wrote the manuscript. GT reviewed and wrote the manuscript. All authors contributed to the article and approved the submitted version.

## Funding

Funding for this research was provided by Fondazione Cariplo, Association for International Cancer Research (AICR no. 09-0713 to GT), Associazione Italiana per la Ricerca sul Cancro (AIRC Special Program Molecular Clinical Oncology, 5 per mille no. 9965 to GT), Multiple Myeloma Research Foundation (MMRF Research Fellow Award to MF), and Ministero della salute (GR-2011-02351686 to MF).

## Conflict of Interest

The authors declare that the research was conducted in the absence of any commercial or financial relationships that could be construed as a potential conflict of interest.
